# Medical History from the Earliest Times

**Published:** 1893-04-08

**Authors:** E. T. Withington


### Medical History from the Earliest Times.

By E. T. Withington, M.A., M.B. Oxon.

### XLIX.—SIMILARS, SYMPATHY, AND SIGNATURES.

We may conveniently pause here to consider three important and closely-allied medical doctrines, first clearly formulated by Basil Valentine and Paracelsus, those of "similars," "sympathy," and "signatures." The germ of these theories may be seen in such primitive beliefs as that the sight of a yellow bird will cure jaundice, and in the ancient legend of the spear of Achilles, which healed the wounds it inflicted. The use of similars had been sanctioned in special cases by Hippocrates, and is, indeed, a natural result of his doctrine of the "vis medicatrix," for if some symptoms in disease are due to the efforts of Nature towards a cure, it is the physician's obvious duty to assist those efforts by means of drugs which produce similar symptoms. Analogous theories may be traced throughout the Middle Ages both among Arabs and Christians. Thus the free-thinking philosopher, Averroës, writes (Colliget v. 19), "Nature has so arranged that diseased organs are benefited by parts similar to them, *e.g.*, in disorders of the stomach the stomachs of animals are commended, especially those of fowls, and for a diseased lung the lungs of animals, especially foxes." So, too, the blessed Albertus Magnus tells us, "It is no secret how every 'like' aids, confirms, loves, acts upon, and embraces that which is like it (quomodo omne simile adjuvet et confirmet suum simile, diligat et moveat et amplectatur illud). And the physicians have already asserted and verified this for their part, for they declare that liver is good for the liver, and every organ for its like." Arnald of Villanova recommends that all soldiers wounded in battle should drink a decoction of persicaria or water-pepper, and that a powder of the same should be sprinkled on the wound, a prescription apparently based on the fact that the plant has a reddish stalk and flower, and leaves spotted, as it were, with blood. These, at least, are the reasons adduced by Paracelsus, who repeats the recommendation. Similarly the leaves of the asarum and the cyclamen were supposed to resemble the human ear, and preparations of those plants were, as we have seen, prescribed in aural diseases by the most distinguished and orthodox practitioners.

But it was the medical mystics who first converted these beliefs into fundamental principles of practice, for they combined in their philosophy two doctrines which especially favoured them—the Neo-Platonic theory that the human body has a mysterious correspondence and sympathy with the outer world, is in fact a little universe or microcosm, and the Christian dogma that all things were created with a view to mankind In one of her visions St. Hildegard heard a voice from heaven which told her that man contains in his body the images or signatures of all things, and in her medical writings she declares that the fact that the mole casts out the bad earth from its burrow in heaps is a sign that the animal when boiled, or dried and powdered, will expel evil humours from the body and cure scrofulous swellings, with much else of a similar nature. Basil Valentine, however, is led to his doctrine of similars chiefly by his anxiety to prove that poisons are medicines. They were created for man; they certainly are not good for the healthy, therefore they are good for the sick. Disease may be likened to a poison which may either be driven out by contraries or drawn out by similars, and the latter is by far the best method and causes least disturbance. He supports this theory by many arguments and examples. It is well known, he says, that if a venomous toad be dried and powdered, and the powder sprinkled on a poisoned wound, it will draw out the poison as a magnet draws iron, and the wound will heal. Soap is a fat which has been treated chemically, and has thereby acquired the power of extracting fat stains from linen; similarly, antimony is a poison, but if treated chemically it will acquire the power of drawing out poisons from the human body, or, in other words, of curing disease. It is interesting to find Paracelsus using exactly the same analogy, in almost the same words, to support his doctrine of *similia similibus*.

But the doctrine of Paracelsus may, perhaps, be more correctly expressed by *analoga analogis*. The Galenic theory of contraries, or of treating hot by cold, &c., he declares is, and always has been, false; the true opposition is between the disease and the arcanum, or remedy which corresponds to it. These he compares to two combatants who are alike hot or cold, and armed with similar weapons, defensive and offensive, "for it is thus that Nature wills her battles should be fought." "So heart cures heart, spleen spleen, lungs lungs; not sow's heart, not cow's spleen, not goat's lungs, but member to corresponding member of the great and the inner man (*i.e.*, the macrocosm and the microcosm)." These correspondencies are shown by signs or signatures, especially in colour. "If a plant has a flower of two colours it has two virtues; if three, three, &c." The spines of the thistle indicate that a decoction of that herb is good for pains in the side; persicaria, for the reasons given above, is the remedy for bleeding wounds; but the leaves and kernels of the peach-tree are also useful, "for see now on the fruit of the peach, if it is pressed by the finger, hollow places remain, so also severe wounds leave hollow places behind them." Lizards are good for anthrax or carbuncle, as is proved by their colour, and the frog is a specific for plague. "Why is the frog so strangely made except that he should be a medicine for the plague? Therefore he has his signature thereto; for see as disgusting (abscheulich) as is the plague, so disgusting is the frog also. When a plague is coming frogs have black spots on their tongues; also so many frogs as sit upon one another at an unusual time, *e.g.*, ten or twenty upon one another, show how many human beings shall be thrown upon one another in one grave. For signatures are to be sought, not only in shape and colour, but also in actions.' Paracelsus proceeds, with his usual strength of language, to declare that those who profanely doubt this make God a liar, for it is He who creates medicines out of the earth, and marks them so that we may recognise their uses.

Basil Valentine was a man of far more sober and scientific mind than Paracelsus, but the following example shows to what depths of absurdity even he could be brought by the spirit of mysticism. Lead, in the language of alchemy, corresponds to Saturn, the slow-moving melancholy planet. Therefore, according to Basil, the salts of lead will attract to themselves by sympathy the humours which cause melancholia, and so cure that disorder. Such theories were considered by the mystics peculiarly profound glimpses into the secrets of Nature, and the less they were connected with common sense, the more they were held to be due to some semi-divine revelation, and the more vigorously did they abuse those who rejected them.

